# Impact of digital infrastructure construction on the migrants’ utilization of basic public health services in China

**DOI:** 10.1186/s12913-024-11221-7

**Published:** 2024-06-23

**Authors:** Haowen Jia

**Affiliations:** School of Economics, Shandong Technology and Business University, Yantai, Shandong China

**Keywords:** Digital infrastructure, Broadband infrastructure, Migrant Health services, Basic Public Health Services, Health Care Access disparities

## Abstract

**Background:**

Global digitalization significantly impacts public health by improving healthcare access for marginalized populations. In China, socioeconomic disparities and the Hukou system create significant barriers for the migrant population to access basic public health services (BPHS). This study aimed to assess how digital infrastructure construction (DIC) affects BPHS utilization among China’s migrant populations, filling a gap in the literature regarding the relationship between digital advancements and health service accessibility.

**Methods:**

This research used micro-level data from the 2018 China Migrants Dynamic Survey and incorporated variables aligned with the Broadband China policy to employ a comprehensive empirical strategy. It included baseline regressions, robustness checks through propensity score matching and machine learning techniques, and heterogeneity analysis to explore the differential impacts of DIC based on gender, age, education level, and Hukou status.

**Results:**

The findings revealed that DIC significantly enhances the likelihood of migrants establishing health records and registering with family doctors, demonstrating quantifiable improvements in health service utilization. Heterogeneity analysis further indicated that the beneficial impacts of DIC were more pronounced among female migrants, those with higher education levels, younger populations, and urban Hukou holders.

**Conclusions:**

DIC plays a crucial role in bridging the accessibility gap to BPHS for migrant populations in China, contributing to narrowing health disparities and advancing social equity. These results emphasize the significance of digital infrastructure in public health strategies and offer valuable insights for policymakers, healthcare providers, and researchers. Future research should prioritize longitudinal studies on the sustained effects of DIC and tailor digital health initiatives to meet the unique needs of migrant populations, promoting inclusive health policy planning and implementation.

## Background

Globally, expanding digital infrastructure—especially improved broadband and internet access—has significantly transformed public health service delivery and accessibility [1]. This transformation is evident in the widespread adoption of telemedicine, electronic health records, and online health information platforms, which collectively work to bridge the gap between healthcare providers and populations, including those in remote or underserved areas [2, 3]. These digital advancements have a global impact, providing unprecedented opportunities to enhance health outcomes and reduce disparities [4, 5]. Against this backdrop, the current study focuses on China—a country at the forefront of digital infrastructure expansion—to explore how such developments specifically influence the accessibility of basic public health services (BPHS) for its migrant population [6]. Placing this research in the broader context of global digitalization trends highlights the critical importance of understanding the connection between digital infrastructure and public health, particularly when addressing the unique challenges encountered by transient and often marginalized communities [7].

In developing countries, the narrative on digital infrastructure and public health is dualistic, encompassing substantial challenges and unprecedented opportunities. Socio-economic disparities, rural-urban divides, and varying technological literacy levels exacerbate health inequities, limiting vulnerable populations’ access to essential health services and information [1]. Digital innovations—encompassing telehealth, electronic health records, and mobile health applications—offer a transformative opportunity to overcome traditional barriers in health service delivery [8]. Research across diverse developing contexts consistently emphasizes the positive impact of digitalization on public health outcomes. Improved connectivity enhances access to health information, elevates patient engagement, and optimizes healthcare delivery efficiency [9]. Such findings lay a compelling groundwork for scrutinizing China’s rapid digital infrastructure evolution, mainly through initiatives like the Broadband China policy, which could unlock new public health advancements, especially for the substantial migrant population [2, 3].

BPHS in China encompasses a range of essential health services provided free or at low cost to the population, including health education, vaccination, maternal and child health services, communicable disease prevention, and chronic disease management. These services are fundamental to maintaining public health and are particularly crucial for vulnerable populations. China’s migrant populations encounter notable challenges in accessing BPHS, influenced by their high mobility, socioeconomic status, and the constraints of the Hukou (household registration) system [10]. The Hukou system is a residency registration system that ties access to various social services, including healthcare and education, to one’s registered place of domicile. Migrants, often holding rural Hukou, move to urban centers for better employment opportunities but are excluded from urban social services and benefits. This exclusion places migrants at a distinct disadvantage in accessing healthcare, as they are less likely to establish health records or register with family doctors, limiting their access to essential health services.

Frequent relocations in migrant life disrupt ongoing medical treatments and hinder the establishment of long-term healthcare relationships. Lower socioeconomic standing often translates into reduced awareness and use of existing health services, as financial constraints and lack of health education hinder their ability to seek and utilize BPHS [11, 12]. This situation has resulted in significant health disparities, as documented in prior research. It highlights a clear gap in the public health infrastructure’s ability to meet the needs of migrant populations effectively [13–15]. To address these disparities, targeted interventions must account for the unique challenges faced by migrants, ensuring equitable access to BPHS regardless of an individual’s Hukou status.

Against this backdrop, the role of digital infrastructure construction (DIC) in mitigating these disparities has become increasingly significant. Improving digital access and literacy through DIC holds can help overcome the unique hurdles migrant workers encounter in accessing BPHS [16]. Improved digital connectivity facilitates more effective communication between migrants and healthcare providers, enables remote consultations, and simplifies health information dissemination and access to personal health records [17]. Existing literature often broadly assesses the impact of DIC on public health outcomes, but it tends to overlook its direct influence on BPHS utilization among migrants [18]. This study aimed to bridge this research gap by specifically investigating how DIC advancements can improve health service accessibility for migrants. By doing so, it underscores the vital role of digital solutions in overcoming systemic barriers faced by migrants. It advocates for inclusive health policy planning and implementation that harnesses technological advancements to ensure equitable healthcare access [19].

This study set out to critically evaluate the impact of DIC on the access and utilization of BPHS by China’s migrant populations amidst the country’s swift digitalization. It aimed to unravel the complexities of how digital infrastructure advancements enhance or impede the ability of migrants to access crucial health services, with a particular focus on the ensuing effects on their health outcomes. This research explored the diverse impacts of DIC and examined how factors such as gender, age, education level, and Hukou status influence the relationship between DIC and BPHS utilization. This comprehensive analysis aimed to illuminate how digital advancements impact health service accessibility across diverse migrant demographics. It aimed to guide policy adjustments and sensitive digital health interventions tailored to the needs of these communities.

The contributions of this investigation are expected to significantly advance the existing body of knowledge on digital infrastructure, public health, and social equity. Recent studies emphasize how digital health interventions can transform healthcare access and outcomes, especially for marginalized populations [6, 20]. This study builds on these findings by specifically examining and identifying the specific impact of DIC on the utilization and access of BPHS among migrant groups in China [2, 7]. It addresses a critical gap in the literature and provides empirical insights into how digital advancements can reduce access disparities in healthcare. These insights are particularly valuable for policymakers, healthcare providers, and researchers, as they underscore strategic avenues for employing digital infrastructure to improve public health outcomes.

Public health policy studies stress integrating digital solutions into existing healthcare frameworks to improve service delivery and efficiency [1, 5]. This research contributes to this body of knowledge by providing empirical evidence on the positive effects of DIC on BPHS utilization, thus supporting the argument for increased investment in digital infrastructure as a public health strategy [9, 21]. Regarding immigrant healthcare access, existing literature highlights various barriers faced by migrants, such as socio-economic disparities and restrictive policies [10, 14]. This study adds to this discourse by highlighting how digital infrastructure can mitigate some of these barriers, offering a viable pathway to more equitable healthcare access for migrant populations [15, 17]. This research fills existing gaps and enriches the discussion on digital health and public health policy by engaging with relevant studies showcasing its valuable contribution to existing knowledge. Ultimately, the research delineates the potential of digital infrastructure to make health services more accessible to marginalized populations, guiding the creation of digital health policies and programs that cater effectively to the varied needs of the population and steering toward the broader goal of attaining health equity.

## Methods

### Data source

This study used micro-level data from the 2018 China Migrants Dynamic Survey (CMDS) conducted by the Chinese National Health Commission. The CMDS targets the migrant population, encompassing information on individual and family characteristics. Notably, it includes detailed data pertinent to the utilization of BPHS, which was the focus of our research [16, 22]. The dataset, spanning nearly all provinces in Mainland China, ensures national representativeness and serves as a robust empirical foundation for analyzing digital infrastructure’s impact on BPHS accessibility for migrant populations [23, 24].

When investigating digital infrastructure development, this study incorporated data that was aligned with the representative Broadband China policy. Launched by the Chinese government on August 17, 2013, this strategic initiative aims to significantly advance China’s broadband infrastructure, highlighting broadband as crucial for economic and social development. The policy sets ambitious eight-year goals for urban and rural broadband expansion, with 2015 and 2020 milestones to achieve widespread connectivity and broadband penetration. Notably, pilot projects initiated between 2014 and 2016 in cities and regions across China served as critical phases in the policy’s execution. These projects evaluated the feasibility and impact of the policy on a smaller scale, providing essential insights into the broadband development process and its socioeconomic effects. The selection of pilot areas for the Broadband China policy included a diverse range of regions across China, encompassing both developed cities in the eastern region and economically less developed cities in the western region. This inclusive approach ensured that the policy’s implementation represented various socio-economic contexts within the country. Existing literature supports the view that the selection process for the Broadband China policy can be considered random [25–27]. These studies treated the policy as a quasi-random experiment, using the broad and inclusive pilot area selection to address endogeneity concerns. By integrating information from the Broadband China policy’s pilot projects with CMDS data at the prefecture level, the current study constructed the dataset required for regression analysis. After data cleansing, including removing outliers, handling missing values, and tail trimming, the sample size for empirical regression in this research amounted to 117,728, with approximately 60% of the sample being impacted by the Broadband China policy.

### Definition of variables

In this research, the primary independent variable was DIC, labeled as 1 if the respondent’s current city of residence or the inflow city was impacted by the Broadband China policy and 0 otherwise. Two dependent variables were employed to assess the accessibility of BPHS among the migrant population: Establishment of Health Record (EHR) and Registration with a Family Doctor (RFD). A value of 1 was assigned to these variables if the respondent had established a personal health record or registered with a family doctor, respectively, and 0 if not. Results presented in Table 1 reveal that approximately 25.4% of the migrant population in the sample had established health records, while 11.5% had registered with a family doctor, indicating generally low accessibility to BPHS among the migrant population.

To enhance the precision of estimating the impact of DIC on the accessibility of BPHS for the migrant population, this study incorporated a comprehensive set of control variables, following established practices in related literature. These variables encompassed individual characteristics, including gender, age, marital status, educational attainment, household registration type (Hukou), and family attributes, such as family demographic structure, income, and expenditure levels. As highlighted in prior research, these variables were selected based on their demonstrated relevance in influencing health service utilization. Including these controls helped to isolate the specific effect of DIC by accounting for other factors that might influence BPHS accessibility, thereby ensuring a more robust and nuanced analysis.

The detailed definitions and descriptive statistics of these variables are presented in Table 1. The study controlled for individual and family characteristics to isolate the specific effect of DIC on BPHS accessibility, enabling a robust analysis of how digital infrastructure improvements affect public health outcomes for migrants.

**Table 1:** Variables definitions and summary statistics

Variables	Definitions	Mean	S.D.
***Explanatory Variable***			
DIC	1 if the respondent’s inflow area is a pilot city of Broadband China policy, otherwise 0.	0.604	0.489
***Dependent Variables***			
EHR	1 if the respondent has established a health record, otherwise 0.	0.254	0.435
RFD	1 if the respondent has registered with a family doctor, otherwise 0.	0.114	0.318
***Individual Characteristics***			
Gender	1 if the respondent is male, otherwise 0	0.513	0.499
Age	Age of the respondent	37.421	11.109
Age Squared	Square of the respondent’s age	1507.254	932.099
Marital Status	1 if the respondent is married, otherwise 0	0.822	0.381
Education Level	1 if the respondent has a high school education or higher, otherwise 0	0.417	0.493
Han Ethnicity	1 if the respondent is of Han ethnicity, otherwise 0	0.916	0.276
Health Status	1 if the respondent is healthy or basically healthy, otherwise 0.	0.977	0.147
Rural Hukou	1 if the respondent has a rural household registration, otherwise 0	0.682	0.465
Duration Away	Number of years the respondent has been away from their household registration location	6.781	6.043
***Household Characteristics***			
Household Size	Number of people living in the respondent’s household	3.178	1.189
Number of Children	Number of children aged 16 or under in the respondent’s household	0.598	0.490
Number of Elderly	Number of elderly aged 65 or over in the respondent’s household	0.043	0.203
Household Income	Log of the respondent’s household’s annual total income	8.749	0.689
Household Expenditure	Log of the respondent’s household’s annual total expenditure	8.099	0.618

### Empirical strategy

In this study, we assessed the impact of the DIC on the utilization of BPHS by China’s migrant population. Our empirical strategy is encapsulated in the following regression model:

$${BPHS}_{icp}=\alpha +\beta {DIC}_{icp}+\gamma {Controls}_{icp}+{Z}_{p}+{\epsilon }_{icp}$$

where ${BPHS}_{icp}$ denotes the utilization of BPHS by migrant individual $i$ in city $c$ of province $p$, measured through indicators such as EHR and RFD. ${DIC}_{icp}$ represents whether the migrant’s city of residence has been impacted by the Broadband China policy. The coefficient of interest, $\beta$, is indicative of the effect of DIC on BPHS utilization. A significant positive $\beta$ would suggest that DIC has effectively enhanced the migrant population’s use of BPHS. This study employed OLS, or linear regression models, despite the binary nature of the dependent variables. We avoided non-linear models like probit or logit to preserve the interpretability of regression coefficients. In linear models, the coefficients directly represent the change in the probability of the dependent variable occurring due to a one-unit change in the independent variable, simplifying the analysis and communication of results. Furthermore, existing literature supported using linear probability models in similar contexts due to their robustness and straightforward interpretation. However, the potential limitations of OLS in handling binary outcomes are acknowledged. To validate the findings, robustness checks were conducted, incorporating more complex models based on machine learning methods.

The model also included ${Controls}_{icp}$, a set of control variables encompassing individual and household characteristics to account for potential confounders. To rigorously control for regional characteristics and capture unobserved heterogeneity at the provincial level, we introduce province-fixed effects, ${Z}_{p}$, into our model. The error term ${\epsilon }_{icp}$ captured random disturbances that might affect the outcome variable outside the explanatory variables specified in the model. The empirical process was conducted using the **reghdfe** command in Stata 16.0.

## Results

### Baseline regression

Table 2 presents the baseline regression results. Columns (1) to (3) analyze the dependent variable EHR, while Columns (4) to (6) concentrate on RFD. The findings in Column (1) reveal that, when not controlling for individual and household characteristics, DIC increases the likelihood of the migrant population establishing health records by 9.83%. After progressively adding individual and household control variables, Column (3) findings demonstrated that digital infrastructure elevates the probability of establishing health records among the migrant population by 9.71%, which is statistically significant at the 1% level. Column (6) examines the effect of DIC on the likelihood of migrant populations registering with a family doctor after accounting for individual and household characteristics. The results reveal a significant positive effect of digital infrastructure on registering with a family doctor. Specifically, DIC enhances the probability of migrant populations signing agreements with family doctors by 2.61%, and this effect is statistically significant at the 5% level. Table 2 highlights DIC’s considerable role in improving BPHS access for migrants.

**Table 2:** Baseline regression results

Variables	(1)	(2)	(3)	(4)	(5)	(6)
	EHR	EHR	EHR	RFD	RFD	RFD
DIC	0.0983***	0.0967***	0.0971***	0.0256**	0.0252**	0.0261**
	(0.0174)	(0.0173)	(0.0173)	(0.0127)	(0.0127)	(0.0126)
Gender		-0.0308***	-0.0309***		-0.0172***	-0.0170***
		(0.0032)	(0.0032)		(0.0021)	(0.0021)
Age		-0.0040***	-0.0026**		-0.0036***	-0.0029***
		(0.0011)	(0.0010)		(0.0008)	(0.0007)
Age Squared		0.0000***	0.0000***		0.0000***	0.0000***
		(0.0000)	(0.0000)		(0.0000)	(0.0000)
Marital Status		0.0501***	0.0426***		0.0312***	0.0257***
		(0.0064)	(0.0065)		(0.0045)	(0.0049)
Education Level		0.0360***	0.0370***		0.0196***	0.0222***
		(0.0046)	(0.0045)		(0.0033)	(0.0032)
Han Ethnicity		-0.0173*	-0.0166		-0.0159**	-0.0146*
		(0.0104)	(0.0101)		(0.0078)	(0.0076)
Health Status		0.0415***	0.0424***		0.0235***	0.0266***
		(0.0100)	(0.0100)		(0.0074)	(0.0074)
Rural Hukou		-0.0135	-0.0135		0.0024	0.0013
		(0.0097)	(0.0098)		(0.0047)	(0.0048)
Duration Away		0.0015***	0.0015***		0.0003	0.0002
		(0.0004)	(0.0004)		(0.0003)	(0.0003)
Household Size			-0.0014			0.0029
			(0.0022)			(0.0019)
Number of Children			0.0172***			0.0067**
			(0.0044)			(0.0031)
Number of Elderly			0.0350***			0.0166***
			(0.0072)			(0.0061)
Household Income			0.0035			-0.0046*
			(0.0033)			(0.0025)
Household Expenditure			-0.0095**			-0.0045
			(0.0047)			(0.0035)
Province FE	YES	YES	YES	YES	YES	YES
Observations	117,728	117,728	117,728	117,728	117,728	117,728
R-squared	0.0866	0.0916	0.0921	0.0796	0.0827	0.0832

Notes: Standard errors, clustered at the city level, are in parentheses. * indicates significance at the 10% level, ** indicates significance at the 5% level, and *** indicates significance at the 1% level

### Robustness checks

Firstly, to account for potential disparities in individual and household characteristics between samples affected by DIC and those not affected, this study conducted propensity score matching (PSM) on the samples used in the baseline regression. This approach ensures that the samples influenced by DIC and those unaffected are comparable in terms of individual and family characteristics, thereby enabling a more accurate analysis of DIC’s impact on the utilization of BPHS by the migrant population. The study specifically calculated propensity scores based on individual and household characteristics, including Gender, Age, Age Squared, Marital Status, Education Level, Han Ethnicity, Health Status, Rural Hukou, Duration Away, Household Size, Number of Children, Number of Elderly, Household Income, and Household Expenditure. Subsequently, the study performed one-to-one nearest neighbor matching, and the regression results for the matched samples are presented in Table 3.

**Table 3:** Propensity score matching method

Variables	(1)	(2)	(3)	(4)	(5)	(6)
	EHR	EHR	EHR	RFD	RFD	RFD
DIC	0.0990***	0.1006***	0.1004***	0.0265**	0.0269**	0.0267**
	(0.0172)	(0.0172)	(0.0172)	(0.0129)	(0.0129)	(0.0128)
Individual characteristics		YES	YES		YES	YES
Household characteristics			YES			YES
Province FE	YES	YES	YES	YES	YES	YES
Observations	46,860	46,860	46,860	46,860	46,860	46,860
R-squared	0.0857	0.0908	0.0913	0.0789	0.0820	0.0825

Notes: Individual characteristics include Gender, Age, Age Squared, Marital Status, Education Level, Han Ethnicity, Health Status, Rural Hukou, and Duration Away. Household Characteristics include Household Size, Number of Children, Number of Elderly, Household Income, and Household Expenditure. Standard errors, clustered at the city level, are in parentheses. * indicates significance at the 10% level, ** indicates significance at the 5% level, and *** indicates significance at the 1% level

Even after improving sample comparability through the PMS method, the significant facilitative effect of DIC on accessing BPHS remained consistent compared to the baseline regression outcomes. This is evident in Columns (3) and (6), where, after controlling for individual and family characteristics, the impact coefficients of DIC on EHR and RFD are 10.04% and 2.67%, respectively, significant at least at the 5% level. These findings affirm the robustness of the baseline regression results.

Secondly, this study modified the key dependent variables measuring the use of BPHS by the migrant population. In the baseline regression, whether respondents had established a personal health record and whether they had signed up with a family doctor were used. For this part of the robustness checks, the definitions of these two variables were altered to include whether the respondents had established or signed up for these services and whether they had heard of them. This adjustment emphasizes whether DIC effectively disseminates BPHS-related information. Results reported in Table 4, particularly in Columns (3) and (6), indicate that after controlling for individual and family characteristics, DIC significantly enhanced the migrant population’s ability to access information regarding establishing health records and signing up with family doctors. In other words, DIC not only directly improves the utilization of BPHS by the migrant population but also enhances their capacity to obtain relevant information about these services.

**Table 4:** Modification of variables definitions

Variables	(1)	(2)	(3)	(4)	(5)	(6)
	EHR	EHR	EHR	RFD	RFD	RFD
DIC	0.0889***	0.0818***	0.0818***	0.0633***	0.0553***	0.0539***
	(0.0175)	(0.0176)	(0.0177)	(0.0158)	(0.0159)	(0.0159)
Individual characteristics		YES	YES		YES	YES
Household characteristics			YES			YES
Province FE	YES	YES	YES	YES	YES	YES
Observations	117,728	117,728	117,728	117,728	117,728	117,728
R-squared	0.0664	0.0751	0.0758	0.0508	0.0625	0.0632

Notes: Individual characteristics include Gender, Age, Age Squared, Marital Status, Education Level, Han Ethnicity, Health Status, Rural Hukou, and Duration Away. Household Characteristics include Household Size, Number of Children, Number of Elderly, Household Income, and Household Expenditure. Standard errors, clustered at the city level, are in parentheses. * indicates significance at the 10% level, ** indicates significance at the 5% level, and *** indicates significance at the 1% level

Thirdly, this study employed machine learning methods to assess the robustness of the baseline regression results. In the baseline analysis, the relationship between DIC and the migrant population’s access to BPHS was assumed to be linear, which may not have fully captured the complexity of the real-world scenario, potentially leading to model misspecification. To address this, the study applied cutting-edge techniques, including LightGBM, LASSO, and Random Forest algorithms, coupled with DML for estimation [28–30]. These techniques model non-linear relationships and interactions between variables, improving data representation. Additionally, machine learning methods such as LightGBM and Random Forest are particularly effective in handling high-dimensional data and capturing complex patterns that traditional linear models might miss. The use of these advanced techniques ensured that the results were robust and reliable. The regression model includes quadratic and cubic terms for individual and household characteristics and utilizes 5-fold cross-validation in the validity tests. These procedures are implemented using the **doubleml** command in Python.

The outcomes presented in Table 5 indicated that, regardless of the specific algorithm employed, the positive impact of DIC on enhancing access to BPHS for the migrant population persisted, as evidenced by the positive coefficients for DIC on both EHR and RFD across Columns (1) to (6), significant at the 1% level. Compared to the baseline regression findings, the results obtained through the DML approach exhibited only minor variations in coefficient magnitude, affirming the robustness of the baseline regression outcomes.

**Table 5:** Double/debiased machine learning method

Variables	(1)	(2)	(3)	(4)	(5)	(6)
	EHR	EHR	EHR	RFD	RFD	RFD
DIC	0.0886***	0.0897***	0.0843***	0.0233***	0.0238***	0.0184***
	(0.0025)	(0.0025)	(0.0072)	(0.0018)	(0.0019)	(0.0052)
Individual characteristics	YES	YES	YES	YES	YES	YES
Household characteristics	YES	YES	YES	YES	YES	YES
Province FE	YES	YES	YES	YES	YES	YES
Algorithm Type	LightGBM	LASSO	Random Forest	LightGBM	LASSO	Random Forest
Observations	117,728	117,728	117,728	117,728	117,728	117,728

Notes: The models include quadratic and cubic terms of individual and household characteristics. Individual characteristics include Gender, Age, Age Squared, Marital Status, Education Level, Han Ethnicity, Health Status, Rural Hukou, and Duration Away. Household Characteristics include Household Size, Number of Children, Number of Elderly, Household Income, and Household Expenditure. Standard errors, clustered at the city level, are in parentheses. * indicates significance at the 10% level, ** indicates significance at the 5% level, and *** indicates significance at the 1% level

**Fig. 1:**
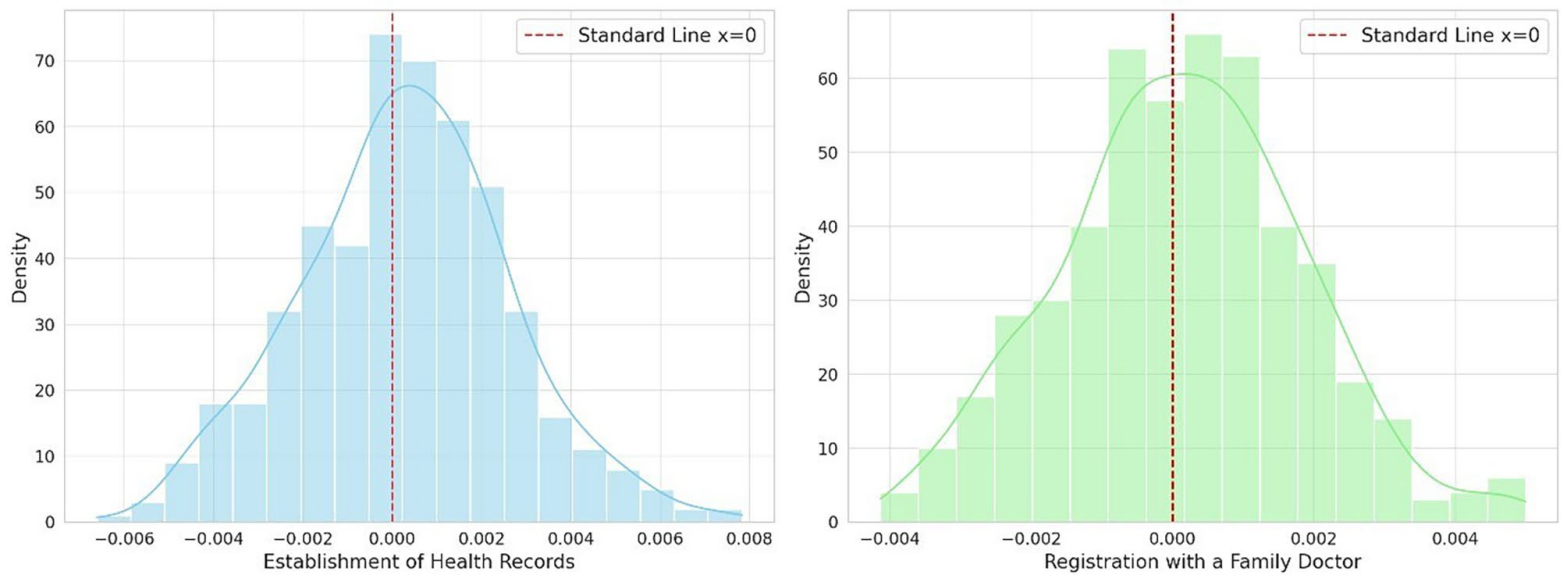
Placebo test

Finally, the robustness of the baseline regression results was further substantiated through a placebo test. The test randomly assigned DIC treatment to samples, mirroring the 60.4% policy impact. This random allocation was then used to conduct regressions, and the coefficients obtained from these regressions were recorded. This process was iterated 500 times to produce a distribution of coefficients, which were subsequently visualized as density plots. This method ensures that the observed impact of DIC is not due to chance but represents a genuine effect by centering the randomly assigned treatment effect around zero. Figure 1 illustrates these results. The left blue graph corresponds to the dependent variable EHR, while the right green graph represents RFD. The red vertical lines represent the standard line where the coefficient equals zero. As illustrated, the effect coefficients for DIC on both EHR and RFD post-randomization clusters are around the zero line. This clustering indicates that the placebo tests have been passed. This convergence around zero confirmed the robustness of the baseline regression findings.

### Heterogeneity analysis

The study performed subgroup regressions to assess DIC’s impact on BPHS accessibility for migrant populations. The results are shown in Table 6, Panels A through D. Firstly, when disaggregating by gender in Panel A, the study found that DIC’s facilitative effects on EHR and RFD were more pronounced and significant for female migrants than their male counterparts. The coefficients for EHR and RFD were larger for females, with higher significance levels. This disparity may stem from gender differences in health-seeking behaviors and digital literacy rates, where women might engage more actively with health services when given digital access, or it could reflect a higher prioritization of health within female migrant subpopulations.

**Table 6:** Heterogeneity analysis

Variables	(1)	(2)	(3)	(4)
	EHR	RFD	EHR	RFD
***Panel A***	***Female Sample***		***Male Sample***	
DIC	0.1008***	0.0307**	0.0939***	0.0220*
	(0.0178)	(0.0127)	(0.0174)	(0.0130)
Observations	57,218	57,218	60,510	60,510
R-squared	0.0977	0.0851	0.0852	0.0803
***Panel B***	***Higher Education Sample***		***Lower Education Sample***	
DIC	0.1143***	0.0398***	0.0872***	0.0178
	(0.0181)	(0.0142)	(0.0177)	(0.0129)
Observations	49,205	49,205	68,523	68,523
R-squared	0.0975	0.0903	0.0873	0.0789
***Panel C***	***Younger Sample***		***Elder Sample***	
DIC	0.1036***	0.0288**	0.0913***	0.0236*
	(0.0183)	(0.0125)	(0.0170)	(0.0134)
Observations	58,755	58,755	58,973	58,973
R-squared	0.1002	0.0918	0.0873	0.0768
***Panel D***	***Urban Hukou Sample***		***Rural Hukou Sample***	
DIC	0.1456***	0.0488**	0.0784***	0.0167
	(0.0212)	(0.0200)	(0.0191)	(0.0133)
Observations	37,359	37,359	80,369	80,369
R-squared	0.0975	0.0769	0.0921	0.0881
Individual characteristics	YES	YES	YES	YES
Household characteristics	YES	YES	YES	YES
Province FE	YES	YES	YES	YES

Notes: Individual characteristics include Gender, Age, Age Squared, Marital Status, Education Level, Han Ethnicity, Health Status, Rural Hukou, and Duration Away. Household Characteristics include Household Size, Number of Children, Number of Elderly, Household Income, and Household Expenditure. Standard errors, clustered at the city level, are in parentheses. * indicates significance at the 10% level, ** indicates significance at the 5% level, and *** indicates significance at the 1% level

Secondly, as shown in Panel B, DIC appeared to have a differential impact based on the educational level of the migrant population. Those with higher education levels (having completed high school or above) showed a more substantial and significant effect on EHR and RFD, with coefficients of 11.43% and 3.98%, respectively. In contrast, the effect was smaller and less significant for those with lower education levels. More educated individuals, with higher digital literacy and better health information comprehension, effectively use digital platforms for health services.

Thirdly, Panel C examined age-based differences, dividing the sample by the median age of 36. The results suggested that younger migrants benefited more significantly from DIC when accessing BPHS, as indicated by larger coefficients for EHR and RFD than the elder cohort. Younger individuals may be more tech-savvy and adaptable to digital services, thereby reaping greater benefits from enhancements in digital infrastructure for health services.

Lastly, Panel D explored the impact of DIC across different household registration types, comparing urban to rural Hukou holders. The results indicated that urban Hukou holders experienced a markedly higher and more significant impact on both EHR and RFD, with coefficients of 14.56% and 4.88%, respectively. This could be due to urban migrants having better access to digital resources and infrastructure or a more significant presence of public health initiatives in urban areas, which were complemented by the digital advancements under the DIC initiative.

## Discussion

This study sheds light on the crucial role of DIC in enhancing migrant populations’ access to BPHS in China. Key findings reveal that DIC significantly boosts the likelihood of migrants establishing health records and registering with family doctors. These results align with existing literature, emphasizing that digital infrastructure is pivotal in determining health service accessibility [5, 7, 8, 31]. However, this study diverges from previous research by quantifying the influence of digital infrastructure on specific aspects of health service utilization—an area less explored [1]. The novel insights this research offers, including a detailed analysis of health record establishment and family doctor registration, contribute to a more nuanced understanding of how digital advancements translate into practical health service improvements for migrants [21, 32–34]. These findings underscore the critical importance of integrating digital infrastructure development with public health strategies to bridge access gaps—an innovative contribution to the current academic discourse.

The empirical findings of this research yield actionable policy recommendations to leverage digital infrastructure to bridge the accessibility gap in health services for migrant populations. Policymakers and healthcare administrators should prioritize expanding digital networks and integrating health services with digital platforms to enhance reach and quality of care [34]. Practically, this involves investing in widespread broadband connectivity, especially in areas with high migrant density [8], and promoting digital literacy programs that enable migrants to use online health resources effectively [35]. Additionally, developing user-friendly digital health portals tailored to migrant needs can facilitate their engagement with health services, including scheduling appointments with family doctors or accessing personal health records [5]. These measures align with the growing literature emphasizing digital inclusion for equitable health service delivery and provide empirical support for policy discussions on leveraging technology to serve marginalized communities [36].

This study reveals that the intersectionality of gender, age, education, and Hukou status significantly influences the effectiveness of DIC in enhancing BPHS utilization. Specifically, female migrants, younger individuals, those with higher education, and urban Hukou holders benefit notably from improvements in digital infrastructure. These findings align with existing research that recognizes the varying impact of digitalization across different population segments [7, 37, 38]. However, this study goes beyond existing literature by delineating the compounded effects of these sociodemographic factors, providing a nuanced analysis of intersectionality in health service accessibility [5, 31]. These insights are invaluable for designing targeted digital health initiatives that cater to the specific needs of diverse migrant subgroups, promoting a more equitable health service delivery system. Moreover, this approach contributes to the theoretical understanding of digital infrastructure’s heterogeneous impacts and underscores the practical importance of acknowledging intersectionality in health policy implementation [1, 39].

The methodological rigor of this study is underscored by its innovative application of machine learning techniques, including the DML method for robustness checks, coupled with PSM to correct for sample bias. These advanced statistical tools bolster the credibility of the findings and establish a precedent for future research. While acknowledging the inherent limitations of cross-sectional data and the endogeneity issues related to the DIC policy, the study paves the way for longitudinal analyses and instrumental variable approaches to validate the causal relationships further. By integrating machine learning into traditional econometric analysis, as demonstrated in this research, we address gaps in the literature and provide a more sophisticated understanding of policy impacts. This enriches methodological discussions, offering a blueprint for blending predictive analytics with causal inference in social science research.

This study’s implications for rapidly digitizing developing countries could be enhanced by discussing the transferability of findings to other contexts, improving research generalizability. China’s experience leveraging digital infrastructure to improve public health services offers valuable insights for other developing nations with similar aspirations. The findings of this study can be applied to other developing countries currently undergoing rapid digital transformation, such as those in Africa and Southeast Asia, where substantial investments are being made in digital infrastructure to bridge healthcare access gaps. The positive impact of digital infrastructure on healthcare utilization observed in China could potentially be replicated in these regions, provided there is a supportive policy environment and adequate investment in digital literacy and infrastructure.

There are, however, significant differences to consider. Healthcare systems in other developing countries may vary in structure, funding, and governance compared to China. For instance, reliance on private healthcare providers in some countries could influence how digital infrastructure impacts public health services. Additionally, urban-rural disparities, cultural factors, and levels of technological adoption differ across countries, potentially affecting the generalizability of the findings.

Conversely, there are similarities. The presence of marginalized populations, economic constraints, and the overarching need to improve healthcare access through innovative means can serve as common ground. By comparing China’s experience with other nations, this study can highlight unique challenges and shared opportunities in digital health.

## Conclusions

This study rigorously assesses the implications of DIC on the accessibility of BPHS among migrant populations in China, shedding light on the transformative role of digital advancements in public health access. The findings reveal tangible improvements in health service utilization, particularly in establishing health records and engagement with family doctors by migrant communities. By providing concrete evidence of DIC’s direct influence on BPHS utilization, this research addresses a notable gap in the existing literature, enriching discussions on the intersection of digital infrastructure with public health and social equity. These insights are crucial for policymakers, healthcare providers, and scholars, highlighting the strategic value of digital infrastructure in enhancing public health outcomes and reducing disparities. The study calls for the strategic integration of digital technologies in health policy and practice, aiming to provide equitable healthcare access to marginalized groups and charting a course towards broader health equity.

Building on these findings, there is a compelling need for future investigations to explore the long-term effects of digital infrastructure improvements on health service access and outcomes, particularly through longitudinal studies focusing on migrant populations. The study calls for prioritizing digital health initiatives to meet these populations’ unique needs, underlining the necessity for inclusive health policy planning and effective implementation. The study underscores the fundamental right to equitable healthcare access through digital advancements for marginalized communities. It underscores the essential role of digital infrastructure in achieving this aim, not only within the context of China but also as a model for global health initiatives. Through its comprehensive analysis, this research highlights the critical need to leverage technological advancements in forging a more equitable and accessible public health infrastructure worldwide.

## Data Availability

Since September 25, 2023, the China Migrants Dynamic Survey data has no longer been directly accessible for registration and application through the Migrant Population Service Center of the China National Health Commission. However, users with specific data requirements can still request access to the data via data exchange, collaborative projects, and other means from the center. Detailed information can be obtained from the following website: https://www.chinaldrk.org.cn/wjw/#/home.

## Abbreviations

DIC: Digital Infrastructure Construction
BPHS: Basic Public Health Services
CMDS: China Migrants Dynamic Survey
EHR: Establishment of Health Record
RFD: Registration with a Family Doctor
PSM: Propensity Score Matching
DML: Double/Debiased Machine Learning
LightGBM: Light Gradient Boosting Machine
LASSO: Least Absolute Shrinkage and Selection Operator
